# Bacterial kinesin light chain (Bklc) links the Btub cytoskeleton to membranes

**DOI:** 10.1038/srep45668

**Published:** 2017-03-30

**Authors:** Lurlène Akendengue, Sylvain Trépout, Martín Graña, Alexis Voegele, Carsten Janke, Bertrand Raynal, Alexandre Chenal, Sergio Marco, Anne Marie Wehenkel

**Affiliations:** 1 Institut Curie, PSL Research University, CNRS UMR3348, F-91405 Orsay, France; 2 Université Paris Sud, Université Paris-Saclay, CNRS UMR3348, F-91405 Orsay, France; 3 INSERM, U1196, Université Paris Sud, Université Paris-Saclay, F-91405 Orsay, France; 4 Institut Curie, PSL Research University, CNRS UMR9187, F-91405 Orsay, France; 5 Institut Pasteur Montevideo, Unidad de Bioinformática, Mataojo 2020, 11400 Montevideo, Uruguay; 6 Institut Pasteur, Unité de Biochimie des Interactions Macromoléculaires, CNRS UMR3528, 28 rue du Dr Roux, 75724 Paris, France; 7 Institut Pasteur, Plateforme de Biophysique Moléculaire, CNRS UMR3528, 28 rue du Dr Roux, 75724 Paris, France

## Abstract

Bacterial kinesin light chain is a TPR domain-containing protein encoded by the *bklc* gene, which co-localizes with the bacterial tubulin (*btub*) genes in a conserved operon in *Prosthecobacter*. Btub heterodimers show high structural homology with eukaryotic tubulin and assemble into head-to-tail protofilaments. Intriguingly, Bklc is homologous to the light chain of the microtubule motor kinesin and could thus represent an additional eukaryotic-like cytoskeletal element in bacteria. Using biochemical characterization as well as cryo-electron tomography we show here that Bklc interacts specifically with Btub protofilaments, as well as lipid vesicles and could thus play a role in anchoring the Btub filaments to the membrane protrusions in *Prosthecobacter* where they specifically localize *in vivo*. This work sheds new light into possible ways in which the microtubule cytoskeleton may have evolved linking precursors of microtubules to the membrane via the kinesin moiety that in today’s eukaryotic cytoskeleton links vesicle-packaged cargo to microtubules.

Tubulin is one of the major components of the eukaryotic cytoskeleton and assembles as an obligate heterodimer into protofilaments that in turn form microtubules, which are generally made up of 13 protofilaments. Although tubulin has long been considered exclusively eukaryotic, bacterial tubulins (Btubs) exist in several *Prosthecobacter* species^1^. *Prosthecobacter* are gram-negative bacteria and belong to the division of *Verrucomicrobia* within the PVC (*Planctomycetes, Verrucomicrobia* and *Chlamydiae*) superphylum and are ubiquitiously present in soil, but also in aquatic environments^2^,^3^. They all present a single prosthecae (narrow extension of the cell wall) that may be required to decrease sedimentation in aquatic environments^2^. A unique feature of these bacteria is the presence of eukaryotic-like tubulin genes, *btubA* and *btubB*, that show high structural homology with eukaryotic tubulin^4^. BtubA and B assemble just like their eukaryotic counterparts as heterodimers in a GTP dependent manner^1^,^5^ and have been shown to localize to the base of the prosthecae of several *Prosthecobacter* species^6^. The *btub* genes coexist with the bacterial tubulin homologue *ftsZ*^7^, suggesting that they are not replacing FtsZ function, but their physiological role remains unclear. Whereas FtsZ forms individual protofilaments, Btubs seem to assemble in more complex bundle structures involving a different number of protofilaments both *in vitro*^4^,^5^ and *in vivo*^6^.

The *in vitro* assembly of BtubA/B protofilaments has been thoroughly studied^5^,^8^,^9^ but very little is known about a third protein, Bklc (Bacterial kinesin light chain), that is systematically present within the genetic cluster that contains the *btub* genes^10^. Interestingly eukaryotic kinesins are a large family of microtubule motors subdivided into about 50 superfamilies (KIFs)^11^. Kinesins are generally composed of a motor domain and a coiled coil domain. Moreover KIF-specific adaptors can be found all over the kinesin families. Kinesin light chains (KLC1–4) are cargo adaptors for some of the KIF5 superfamily kinesin motors that are involved in axonal transport of synaptic vesicles, membrane organelles, as well as tubulin dimers^11^.

The *btub* and *bklc* genes are co-transcribed and are thought to form a *bona fide* operon^1^,^10^ but a functional link between the Btubs and Bklc has not yet been described. In order to understand whether *btubAB* and *bklc* form a functional operon we set out to characterize Bklc with respect to the bacterial tubulins. Here we show that the 3 proteins do indeed interact when Btubs are polymerized. Moreover Bklc was found to bind to lipid vesicles, and cryo-electron tomography allowed us to visualize a ternary complex between Btub filaments, Bklc and lipids, strongly suggesting a role in the membrane anchorage of the Btub filaments within the prosthecae. This work provides new elements towards understanding possible ways in which the microtubule cytoskeleton may have evolved linking precursors of microtubules to the membrane via the kinesin moiety.

## Results and Discussion

### Bklc characterization

**Figure 1:**
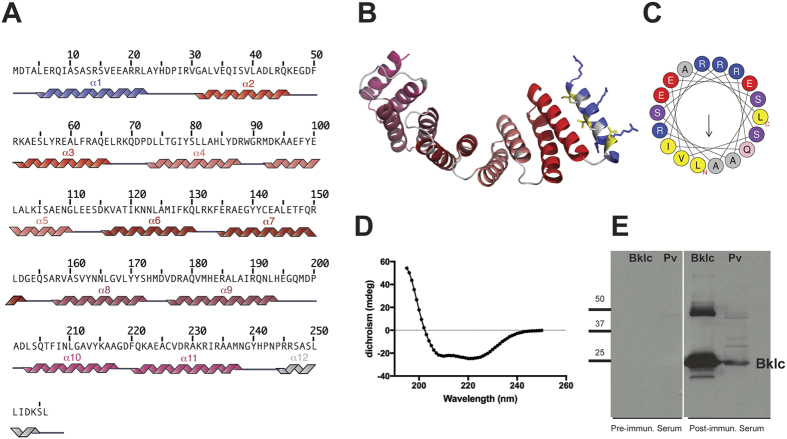
Bklc characterization (**A**). Secondary structure prediction of Bklc from P. vanneervenii (GenBank accession number: CAP16140.1). The 5 predicted TPR domains cover the following sequences: TPR1: 34–67; TPR2: 76–109; TPR3: 118–151; TPR4: 160–193; TPR5: 204–237 (**B**). 3D model of Bklc generated using Phyre2^30^. The N-terminal helix is shown in blue; the positively charged arginine residues are highlighted in blue and the hydrophobic residues in yellow (in accordance with the colors used in Fig. 1C) (**C**). Helical wheel representation of the N-terminal helix generated using Heliquest^31^. The arrow indicates the hydrophobic moment (**D**). Far-UV CD spectrum of Bklc showing a typical profile of an alpha-helical protein (**E**). Bklc is expressed *in vivo* in Prosthecobacter cell extracts. Lanes labeled Bklc correspond to purified recombinant Bklc protein. Lanes labeled Pv correspond to *Prosthecobacter vanneervenii* cell extracts.

Bklc had previously been described as a tetratricopeptide repeat (TPR)-containing protein with homology to eukaryotic kinesin light chain proteins^10^. TPR repeats are ubiquitously present in nature and play a major role in scaffolding and protein-protein interactions^12^. The entire central region of Bklc (residues 30–238) displays significant sequence identity (24%) with the experimental structure of human kinesin light chain HKLC2 (PDB code 3ceq), indicating that the central core of Bklc is an all alpha-helical protein with 5 central TPRs (10 helices, Fig. 1A,B). The additional N-terminal (residues 1–29) and C-terminal (239–256) segments of Bklc also have a high alpha-helix propensity (Fig. 1A) and might correspond to the flanking helices that are thought to have a “capping” or solubilizing function in TPRs^12^. The analysis of the hydrophobic and polar properties of these helices shows an amphipathic character for the N-terminal helix, with a hydrophobic and a polar face (with positive mean net charge, Fig. 1B,C).

Homologues of Bklc are found in all domains of life. This is in contrast with Btubs for which all detectable homologues out of a high similarity zone (*Prosthecobacter* genus) are from eukaryotic organisms. More sensitive profile-based searches confirmed this tendency and we were able to collect a diverse set of distant homologues against non-redundant databases. Although the promiscuity and ubiquity of TPR repeats in nature preclude extracting a clear evolutionary path for the origin of Bklc, some consistent results are noteworthy. First, eukaryotic homologues are found in simple unicellular eukaryotes (including *Ostreococcus tauri*, a marine green alga and the smallest free-living eukaryote) as well as higher eukaryotes. We also confirmed homology to kinesin light chains from vertebrates, including human kinesin light chain as well as the ciliary protein nephrocystin-3 (NPHP3).

In order to biochemically characterize Bklc we produced the C-terminal poly-histidine-tagged recombinant protein from *Prosthecobacter vanneervenii*. Bklc is a soluble protein and could be purified as a monomer using size exclusion chromatography. The secondary structure content of Bklc was analyzed by far-UV circular dichroism spectroscopy, which confirmed the predominantly alpha helical nature of the protein (Fig. 1D). We also wanted to verify that Bklc has a biological relevance and tested for its presence *in vivo* in *P. vanneervenii* cell extracts obtained from late exponential cultures. We produced polyclonal antibodies against recombinant Bklc in order to detect the protein in cell culture extracts. Using Western blot analysis with anti-Bklc antibodies we were indeed able to detect a band specifically recognized by the antibody and corresponding to the size of recombinant Bklc (Fig. 1E). We were thus able to confirm that the *bklc* gene is expressed and the protein present in *Prosthecobacter* cultures.

### Bklc binds to polymerized Btubs

**Figure 2:**
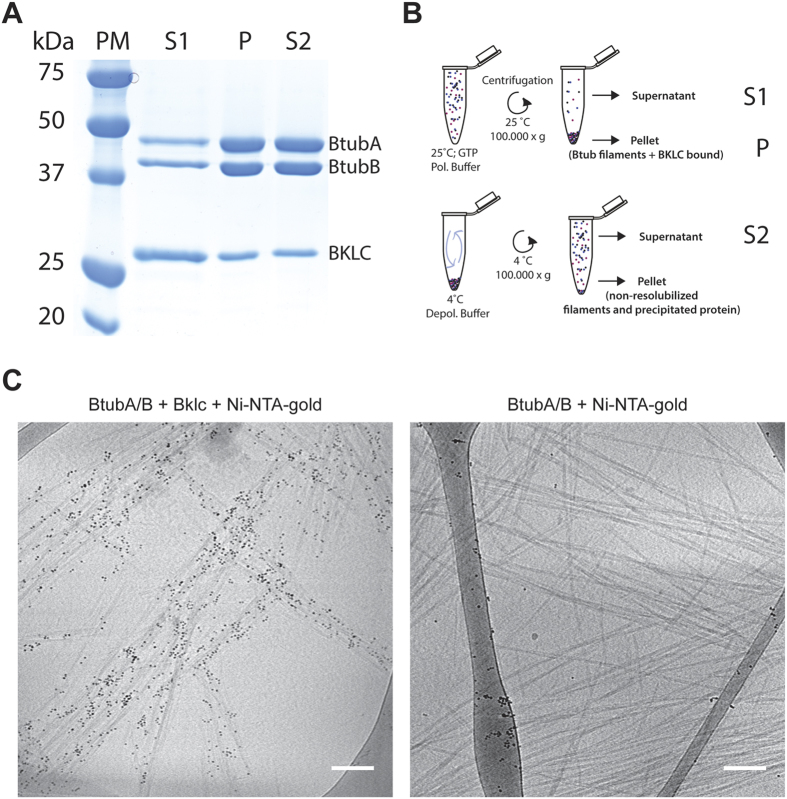
Btub-Bklc interaction (**A**). Coomassie stained SDS-PAGE showing that Bklc reversibly binds to Btub polymers in co-sedimentation assays (**B**). Schematic representation of the experimental procedure corresponding to fractions S1, P and S2 of the co-sedimentation assay (**C**). Ni-NTA-gold labeling of Bklc bound to Btubs in cryo-electron microscopy (left) Btub filaments without Bklc (right) Scale bar: 100 nm.

As *bklc* systematically co-localizes with the *btub* genes we set out to understand whether Bklc is functionally related to the Btubs by testing for the interaction between BtubA/B and Bklc. We could not detect an interaction of Bklc with non-polymerized Btub proteins neither by size exclusion chromatography (Supplementary Figure S1) nor by pull-down experiments suggesting very low or no affinity of Bklc for soluble Btubs. In order to detect binding of Bklc to polymerized Btubs we carried out sedimentation assays. In brief, purified and stoichiometric amounts of BtubA/B (generally 10 μM of the heterodimer) were incubated with an excess of Bklc per Btub monomer (10 or 20 μM) in polymerizing conditions. After high-speed centrifugation the Btub polymers were recovered in the pellet (P) together with Bklc, suggesting an interaction of the latter with the assembled Btub filaments (Fig. 2A). As a further control we tested for the reversibility of the binding (to exclude protein precipitation) by depolymerizing the Btub filaments present in the pellet and recovering the soluble fraction after depolymerization (Supernatant 2 (S2); Fig. 2B). Bklc as well as the Btubs were recovered in the soluble fraction and consecutive co-polymerization cycles could be carried without losing Bklc. Visual inspection of the SDS PAGE bands from the sedimentation assay suggests sub-stoichiometric binding of Bklc to Btub polymers.

Considering the technical difficulties to accurately measure polymer-associated stoichiometry in solution, we used the ImageJ software for densitometric quantification of gel band intensities of Btub and Bklc. A total of 10 μM of BtubA/B was used in all the gels analyzed where BtubA and B were present in stoichiometric amounts as shown by Sontag and colleagues^8^. Bklc was added at a concentration of 10 or 20 μM. We thus quantified the relative intensities for BtubA, BtubB and Bklc from the gel bands (knowing that S1 + P1 correspond to the total amount of protein input) and calculated the intensity that would correspond to the amount of protein loaded. For the same amount of protein, the signal from the Btubs on the Coomassie stained gel was about twice that of Bklc. Relating these values to the S2 fraction of the sedimentation assay, the stoichiometry thus estimated corresponds to about 1 Bklc monomer per BtubA/B heterodimer.

Under the conditions used in our study Bklc interacted with polymerized Btubs but not monomeric or dimeric proteins, suggesting that the binding mode of Bklc may be sensitive to a particular environment only found within the context of the Btub polymer. Several microtubule-associated proteins (MAPs) have been shown to discriminate between assembled microtubules and unpolymerized tubulin. Two structurally characterized examples are the plus end tracking protein EB3^13^ and doublecortin (DXC^14^), both proteins binding between two protofilaments making longitudinal as well as lateral contacts with the tubulin dimers and thus fully relying on the assembled microtubule for binding^15^. A second possibility for Btub-Bklc interaction would be the recognition of a polymer-specific conformation of the Btub heterodimer. In the case of tubulin, it is known that tubulin dimers can adopt different conformations depending on which nucleotide is bound and whether they are incorporated into a microtubule or are free in solution^14^,^16^. While the observed stoichiometry of 1 Bklc per BtubA/B heterodimer would be compatible with both models above, we observed that Bklc binds several BtubA/B filament bundles with different lateral assemblies (that co-exist *in vitro,* see results below), suggesting the recognition of a polymer-associated conformation rather than a polymer specific interface.

To confirm the results obtained from the sedimentation assay and to visualize any potential changes to the Btub filament assembly we carried out cryo-electron microscopy analysis of the assembly. Btubs and Bklc were co-polymerized and Ni-NTA nanogold beads were added just prior to freezing in order to label the poly-histidine tag of Bklc. The Btub filaments in the presence of Bklc were largely decorated by Ni-NTA beads whereas the negative control of Btubs with Ni-NTA beads alone were not (Fig. 2C), further confirming the functional relationship between the 3 proteins of the operon. Using a light scattering assay to follow Btub assembly we could not observe significant changes in assembly or critical concentration in the presence of Bklc (Supplementary Figure S2), suggesting that Bklc does not affect polymerization properties of the Btubs.

**Figure 3:**
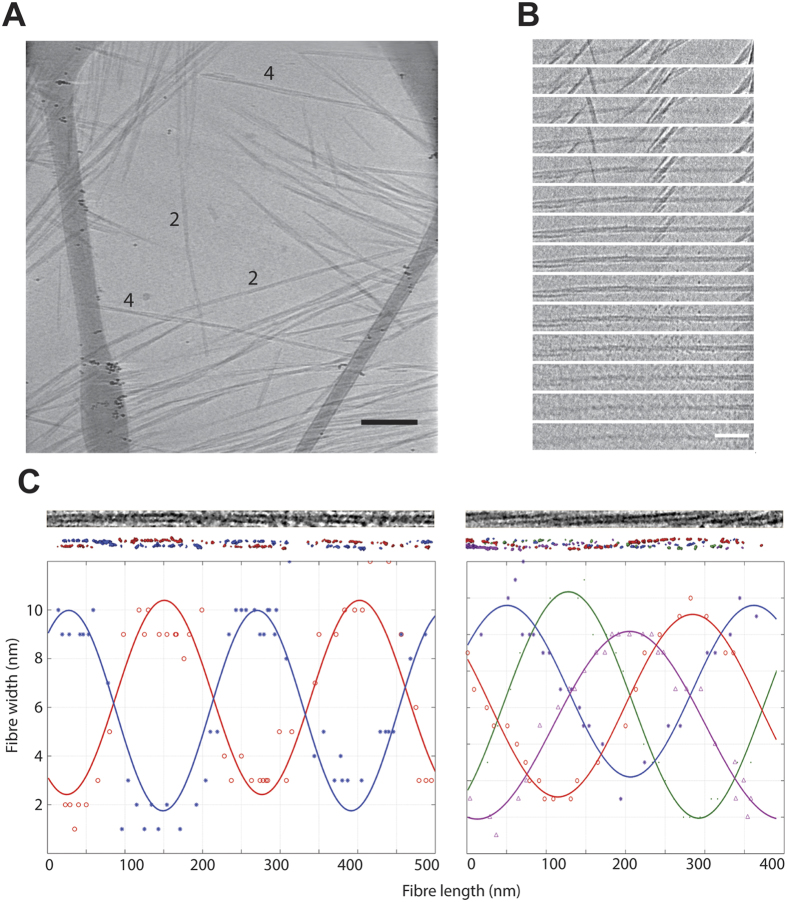
Structural characterization of Btub filaments (**A**). The image shows a 2D projection from a tomogram of BtubA/B fiber (see also Supplementary Movie S4 for corresponding tomogram). Some representative filaments of 2 and 4 are highlighted in the figure. Scale bar: 100 nm (**B**). A sequential view through a tomogram of a 2 filament assembly is shown in Fig. 3C (left) Scale bar: 50 nm (**C**). 3D structures of Btub filament assemblies. Top, experimental images of Btubs extracted from two cryo-tomograms. Middle, rendering of threshold-based manually segmented tomograms showing each filament in a single color (red, blue, violet and green). Bottom, distance (width and length) plot of each filament depicting their periodicity. For the helical reconstruction and periodicity the mean squared error (MSE) for each curve is as follows: Left plot: blue curve (*) = 1.96; red curve (○) = 1.52; Right plot: blue curve (*) = 0.53; red curve (○) = 0.41; green curve (•) = 0.39; purple curve (∆) = 1.01.

Btubs have been suggested to assemble as 5-protofilament bacterial microtubules (bMTs)^6^ *in vivo*, so we asked if Bklc could induce different inter-filament assemblies. Our images of Btub filaments alone did not show evidence for bMTs, in fact all of our polymers appeared to be helical polymers of 2 or 4 protofilaments, as previously seen in negative stain EM^4^,^5^. We analyzed the 3D structures of some representative filament assemblies made up of 2 or 4 filaments and determined their periodicity (Fig. 3 and Supplemental Movie S1 and S2). For double filaments a cross over was observed every 110 to 130 nm corresponding to a periodicity of 250 nm. The helical periodicity increases with 4 filaments to 310 nm suggesting a flattening out as filament numbers increase. Indeed we observed 6 filament assemblies (Supplemental Movie S3), but we were not able to accurately measure the periodicity due to fiber crowding on the images. However, based on half-repeats, which are 370 nm long, we can estimate the periodicity to be around 740 nm. In the presence of Bklc we did not observe any significant changes in the assembly properties of the filaments with respect to each other (Supplementary Movies S4 and S5).

### Purified Bklc binds to lipid membranes

**Figure 4:**
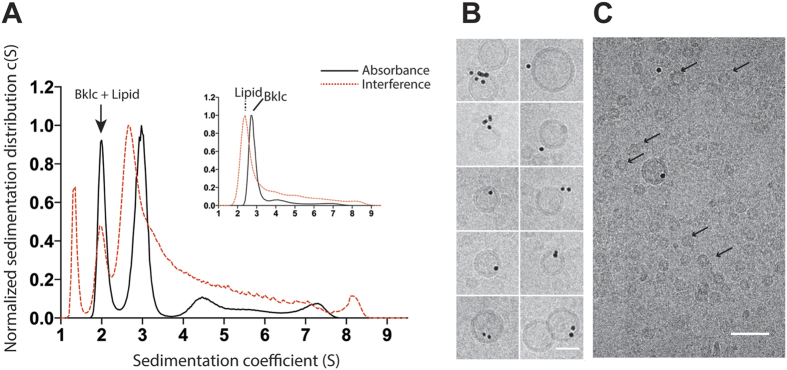
Bklc binding to lipid vesicles (**A**). Sedimentation profiles of Bklc + SUVs (main graph) detected by absorbance at 280 nm and interference; control Bklc alone detected by absorbance at 280 nm (inset, black continuous line) and SUVs alone detected by interference (inset, red dotted line) (**B**). SUV-Bklc co-localization as indicated by the Ni-NTA-gold beads directed against the His-tag of Bklc (scale bar: 30 nm) (**C**). Upon incubation of SUVs with Bklc, small lipid assemblies are formed (some highlighted by arrows) that could correspond to the lowest sedimenting species of Fig. 4A main graph (scale bar: 50 nm).

The above results showed that Bklc directly interacts with Btub filaments but its physiological role remains unclear. The presence of an N-terminal amphipathic helix as well as previous localization studies of Btubs within the base of the prosthecae and near the plasma membrane of *P. vanneervenii*^6^ suggested that Bklc could bind to membranes and play a functional role in membrane tethering of the Btub filaments. To determine if Bklc interacted with membranes, we performed AUC sedimentation velocity experiments. Experiments were carried out using a double optical detection system by absorbance at 280 nm (ABS; specific to proteins such as Bklc) and interferometry (IF) that can detect both protein and lipids. Bklc on its own sediments as a main species with a sedimentation coefficient of 2.8 S and frictional ratio of 1.2 giving a calculated molecular mass of 30.3 kDa in accordance with a monomeric state of the protein (Fig. 4A insert). The small unilamellar vesicles (SUVs) were run separately and showed a main peak at 2.4 S (Fig. 4A insert). Upon incubation of both SUVs and Bklc a new peak appears at 2.0 S with a signal for both Bklc (ABS and IF) and lipids (IF) providing direct experimental evidence for their interaction (Fig. 4A). Integration of absorbance and interference signals showed that the peak contains lipids as well as proteins. The decrease in sedimentation of Bklc from 2.8 S to 2.0 S is in accordance with a higher buoyancy and thus “floatation” of Bklc in interaction with lipid vesicles. We confirmed the Bklc-SUV interaction using cryo-EM where Ni-NTA nano-gold beads that label the C-terminal His-tag of Bklc were co-localized with lipid vesicles (Fig. 4B). An additional new peak was detected by interference only, at a sedimentation value of 1.3 S suggesting the presence of small lipid assemblies. Indeed our cryo-EM data showed that Bklc induces a resizing of lipid vesicles and we observed small objects (about 14 nm; Fig. 4C) that were not found when SUVs were imaged alone. It is likely that these vesicles correspond to the peak of 1.3 S.

**Figure 5:**
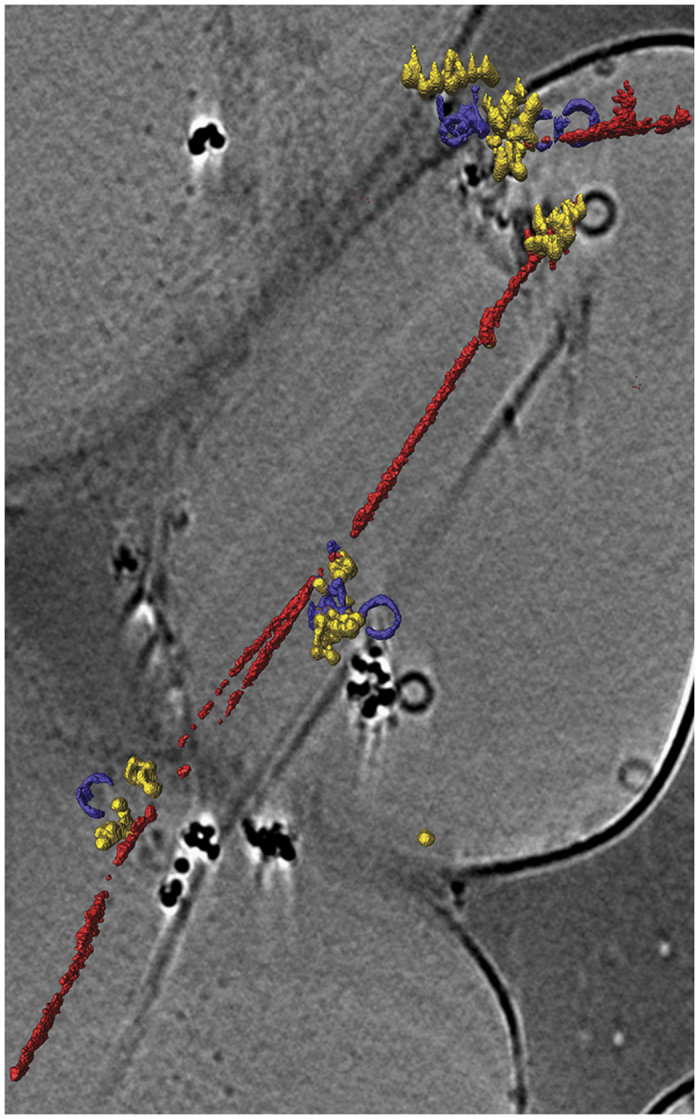
Cryo-EM image and 3D representation of the ternary complex between Btub fibers (red), SUVs (purple) and Bklc + Ni-NTA-Gold (yellow). The 3D iso-surfaces are overlaid on a 2D image corresponding to one z-plane from the tomogram.

Btub filaments have been described in close vicinity to the plasma membrane of *P. vanneervenii*^6^. Our sedimentation and AUC data above suggest that Bklc could therefore tether the Btubs to the membrane. To understand if Bklc could mediate both Btub and SUV interactions simultaneously we incubated BtubAB (5 μM) with Bklc (5 μM) and SUVs (4 mM lipid) in polymerizing conditions for 10 to 20 minutes for subsequent EM imaging. Just prior to flash-freezing the mixture was diluted in polymerization buffer with Ni-NTA nanogold beads. Gold labeling of the Bklc His tag localized the protein to the area between the filaments and the lipid vesicles, indicating that the protein could mediate their interaction. A 3D reconstitution showed the direct interaction of all 3 partners (Fig. 5). Taken together our results point to a physiological role of Bklc as a membrane tether of Btub filaments in *Prosthecobacter*.

From an evolutionary point of view the Btub operon seems to be clearly related to the eukaryotic kingdom from which *Prosthecobacter* have acquired it via horizontal gene transfer. The ubiquitous presence of TPR domains in all domains of life hampers determination of Bklc origin. Yet Bklc is probably a molecular partner since the Btub transfer event. Supporting this hypothesis is the abrupt decay in sequence identity (from >80% to <40%) for the three proteins out of the ‘*Verrucomicrobia*’ zone as well as our functional characterization. Our work shows that Bklc can bind both Btubs and SUVs, leading us to speculate that an early eukaryotic MAP could have been involved in membrane tethering of microtubules. It is interesting to note that kinesin motors were postulated to be present in the last eukaryotic common ancestor (LECA)^17^. One possibility may be that Btub-Bklc complex represents a transition period in eukaryotic tubulin evolution where the microtubule cytoskeleton acquired its complexity. Indeed membrane tethering of microtubules is thought to be an important step in the evolution of the eukaryotic cell, evolving from vesicle binding modules to more sophisticated microtubule-membrane compartment such as the cilium^18^. Interestingly, the second close homologue we found for Bklc in higher eukaryotes is NPHP3, a multi-domain ciliary protein that apart from the TPR domains also includes a tubulin modifying domain (tubulin tyrosine Ligase, TTL) and is thus likely to directly interact with tubulin or microtubules^19^. If our hypothesis were correct the early link between membranes and microtubules would have consisted of a TPR domain-containing protein. TPR domains are helical tandem repeats, that when present as multiple copies, can form alpha solenoids similar to Heat or Armadillo (ARM) domains. The latter are predominantly found in eukaryotes whereas TPRs are widespread in both eukaryotes and prokaryotes^20^. Alpha-solenoid based architectures are evolutionary conserved modules involved in membrane trafficking and shaping^20^ and it is thought that membrane-curving protein modules were required to evolve the endomembrane system^21^.

## Materials and Methods

### Protein Expression and Purification

The *btubA* and *btubB* genes from *P. dejongeii* were co-expressed without an affinity tag in a pHis17 vector from a biscistronic gene construct where the native intergenic region remained intact (gift from JM. Andreu). Transformed *E. coli* C41 (DE3) cells were grown to an optical density (OD_600nm_) of 0.4 at 37 °C and protein expression was induced with 0.25 mM IPTG for 3 hours at 37 °C. Cell pellets were harvested and flash frozen in liquid nitrogen. BtubA and BtubB proteins were purified by an initial cycle of polymerization/depolymerization, followed by size exclusion chromatography. In brief, bacterial cell pellets from 1-liter cultures were resuspended in 20 ml lysis buffer (50 mM Tris-HCl pH 8 supplemented with EDTA-free protease inhibitor cocktails (ROCHE)). Cells were disrupted by sonication and the lysate was centrifuged for 30 min at 30.000 rpm. The cleared lysate was completed with the polymerization buffer (300 mM potassium glutamate, 5 mM MgCl_2_, 2 mM GTP, 1 mM EGTA) and incubated for 10 minutes at 25 °C. The mixture was centrifuged for 30 min at 100,000 × g at 25 °C (in a pre-warmed Ti-70 rotor). The semi-transparent protein pellet (P1) corresponding to the polymerized Btubs was carefully dissolved in a small volume (1.5 ml) of cold depolymerization buffer (20 mM Tris-HCl, 1 mM EGTA, pH 7.5) for 20 minutes on ice to allow for complete depolymerization of the BtubA/B-assembled polymers. The sample was centrifuged 30 minutes at 100,000 × g at 4 °C in a precooled rotor (TLA-55). BtubA and BtubB were now recovered in the supernatant (S2). The supernatant (S2) containing Btub A/B was concentrated and loaded onto a size exclusion column (GE Healthcare - Hiload Superdex 200 PG 16–60) pre-equilibrated at 4 °C in the gel filtration buffer (20 mM Tris-HCl, 1 mM EDTA pH 7.5). The fractions containing BtubA and BtubB were then pooled and concentrated to about 20 mg/ml, flash frozen in liquid nitrogen and stored at −80 °C.

The *bklc* gene from *P. vanneervenii* was expressed from pHis17 with a C-terminal His-tag (gift from M. Pilhofer). Transformed *E. coli* C41 (DE3) cells were grown to an optical density (OD_600nm_) of 0.4 at 37 °C followed by an equilibration at 16 °C for 20 minutes. Protein expression was induced with 0.25 mM IPTG for 20 hours at 16 °C. Cell pellets were harvested and flash frozen in liquid nitrogen, and resuspended in lysis buffer (50 mM Tris-HCl pH 7.5, 500 mM NaCl, 5% glycerol, 1 mM DTT, EDTA-free protease inhibitor cocktail). The resuspension was sonicated, and the lysate was centrifuged for 30 minutes at 30,000 rpm. The cleared lysate was loaded onto a Ni-NTA affinity chromatography column (HisTrap FF, GE Healthcare). Nonspecific proteins were removed by washing the column with buffer A (20 mM Tris-HCl, 200 mM NaCl, 5% glycerol, 1 mM DTT and 10 mM imidazole, pH 7.5). Bklc-His was eluted with a linear gradient of imidazole (Buffer B, 20 mM Tris-HCl pH 7.5, 200 mM NaCl, 5% glycerol, 1 mM DTT, 1 M imidazole). The eluted fractions containing Bklc were concentrated and loaded onto a size exclusion column (GE Healthcare, Superdex 200 10/300) pre-equilibrated at 4 °C in the gel filtration buffer (20 mM TrisHCl pH 7.5, 200 mM NaCl, 5% glycerol 1 mM DTT). The final elution peak was concentrated to 2 mg/ml, flash frozen in liquid nitrogen and stored at −80 °C.

### Circular Dichroism

Far-UV spectra (195–250 nm) of Bklc in solution were obtained by circular dichroism on an AVIV 215 machine. Bklc was used at a concentration of 17.5 μM in its storage Buffer (20 mM TrisHCl pH 7.5, 200 mM NaCl, 5% glycerol 1 mM DTT) in a cuvette with optical path length of 0.2 mm.

### Analyical Ultracentrifugation

Bklc, SUVs, as well as Bklc-SUV complexes at the following final concentration [Bklc] 27 μM, [Lipid] 2 mM were centrifuged at 42 000 rpm in a Beckman Coulter XL-1 analytical ultracentrifuge, at 20 °C in a four-hole AN 60–Ti rotor equipped with 12-mm double-sector epoxy centerpieces. Detection of the protein and SUV concentration as a function of radial position and time was performed by optical density measurements at 280 nm and interferometry. Ultracentrifugation experiments were performed in 25 mM Tris-HCl buffer (pH 7.5) containing 150 mM NaCl. The following parameters were calculated with Sednterp software and used for the results analysis: partial specific volumes 0.729 ml.g^−1^ for Bklc, 0.979 ml.g^−1^ for lipid, viscosity 0.01023 poise, and density 1.0053 g.ml^−1^. Sedimentation velocity data analysis was performed by continuous size distribution analysis c(s) using Sedfit 15.01 software^22^.

### Prosthecobacter cultures

*Prosthecobacter vanneervenii* strain 12252 was purchased from DSMZ (Leibniz-Institut DSMZ-Deutsche Sammlung von Mikroorganismen und Zellkulturen GmbH, www.dsmz.de) and grown in liquid MMB medium as described in DSMZ recipe 628 (https://www.dsmz.de/microorganisms/medium/pdf/DSMZ_Medium628.pdf). Cultures were grown at 26 °C in an orbital shaker and reached a maximal optical density OD_600nm_ = 0.7 after 65 hours of culture. Cell pellets were harvested by extended centrifugation (90 minutes at 20.000 × g).

### Antibody production and Western Blot

Rabbit polyclonal antibodies were generated against recombinant Bklc-His from *P. vanneervenii* by PTL (Pettinghill Technology, London). The specific recognition of anti-Bklc antibodies was validated by testing pre-immunized and different post-immunization bleeds against purified recombinant protein (0.2 μg per lane) used for immunization as well as total cell extracts from *P. vanneervenii* (70 μg total protein per lane). The sera were diluted at a ratio of 1:2000 and HRP-conjugated anti-rabbit secondary antibodies (1:10 000, GE Healthcare NA934) were used for chemiluminescent detection (Luminata Forte, Millipore) on film (Amersham Hyperfilm ECL). The pre-immune sera and anti-Bklc antibody were blotted on separate membranes (from a single gel transferred to a single membrane, and then cut in two). The two membranes were laid side by side to reconstitute the original gel for chemiluminescent exposure on film.

### Sedimentation assay

Purified BtubA/B (generally at a concentration of 10 μM) were polymerized as described in ref. 23. In brief BtubA/B were incubated for 10 minutes at 25 °C in polymerization buffer (20 mM TrisHCl pH 7.5, 300 mM potassium glutamate, 5 mM MgCl_2_, 1 mM EGTA, 2 mM GTP). Polymerized Btub filaments were sedimented into the pellet after ultracentrifugation for 30 minutes at 100.000 × g at 25 °C. In order to check for reversibility of the polymerization, the pellet was resuspended in depolymerization buffer (20 mM TrisHCl pH 7.5, 1 mM EGTA) at 4 °C for 20 minutes. The sample was centrifuged at 100.000 × g for 20 minutes at 4 °C and the depolymerized Btubs were recovered in the soluble fraction. Co-polymerization with Bklc (varying concentrations) was carried out according to the same protocol as Btubs alone.

### Densitometric quantification of gel bands

The SDS-PAGE gels to be analyzed were scanned and loaded into ImageJ software (Rasband, W.S., ImageJ, U. S. National Institutes of Health, Bethesda, Maryland, USA, http://imagej.nih.gov/ij/, 1997–2016). The Images were converted to 32 bit grayscale images and rectangles were drawn around the area of the lanes to analyze and the band intensity was plotted per lane. The peaks corresponding to the band intensities were quantified and the ratios of Btub:Bklc were calculated on an average of 3 independent gels from three independent experiments.

### Small unilamellar vesicle preparation

Small unilamellar vesicles (SUVs) were prepared by reverse phase evaporation^24^ from a chloroform solution made of an 8:2 mixture of 1-palmitoyl-2-oleoyl phosphatidylcholine (POPC) and 1-palmitoyl-2-oleoylglycero-3-phosphoglycerol (POPG) to a final concentration of 10 mM lipids. The chloroform was removed by evaporation in a rotavapor under vacuum. The dried phospholipid film was protected from oxidation by a layer of argon and then resuspended in a mixture of diethyl ether and buffer solution (25 mM TrisHCl, pH 7.6, 150 mM NaCl), followed by sonication to make an emulsion of lipid, ether and buffer. The reverse phase evaporation was performed by slowly evaporating the ether in the rotavapor by slowly decreasing the pressure down to the vacuum to avoid ether boiling. The lipid suspension was then extruded through polycarbonate filters (from 0.8 to 0.2 μm pore sizes) to obtain large unilamellar vesicles (LUVs). The SUVs were obtained by further sonication of the LUV solution. The diameter and charge of SUV were measured by dynamic light scattering (DLS) and electrophoretic mobility on a Zetasizer Nano instrument (Malvern Instruments)^25^

### Sample preparation for electron microscopy

The images obtained for Btub filaments in absence or presence of Bklc were obtained by 2 consecutive cycles of polymerization in order to remove protein noise that could be generated from non-polymerization competent molecules. The Ni-NTA-nanogold beads (5 nm; purchased from Nanoprobes) were added after the second polymerization and used at a final concentration of 50 nM.

For the SUV images with or without Bklc, experiments were carried out at room temperature, and the SUVs were incubated with or without Bklc for 45–60 minutes using concentrations of 2.5 mM and 4 M for SUVs and Bklc respectively in 20 mM TrisHCl pH 7.5 and 150 mM NaCl. The Ni-NTA-nanogold beads (5 nm; purchased from Nanoprobes) were added just prior to sample freezing and used at a final concentration of 50 nM.

For plunge freezing of samples, a 5 μl drop of sample was deposited onto 300 mesh holey carbon copper grids (Ted Pella). The excess of solution was manually blotted using a Whatman filter paper and the grid was plunge-frozen in liquid ethane using a Leica EM-CPC.

### Electron microscopy imaging and data processing

Data acquisition was performed on a 200 kV field emission gun JEOL 2200FS electron microscope equipped with a Gatan US1000 slow scan CCD camera and an in-column energy-filter. Zero-loss (slit: 20 eV) high magnification images were acquired at pixel sizes between 7 and 2 Å at the specimen level with nominal defocus between 1 and 4 μm depending on the experiment. Tilt-series dataset were collected under zero-loss conditions (slit: 25 eV) at a pixel size of 7 Å at the specimen level with nominal defocus varying from 5 to 10 μm depending on the experiment using an in-house developed software.

For data processing of the Btub fibers Tilt-series were aligned and reconstructed using TomoJ v2.32^26^. The alignments were performed using the local minima algorithm^27^ and reconstruction was generated using OS-SART-GPU (iterations: 100, relaxation coefficient: 0.01, volume update: every 4 images).

The periodicity of the helical filament repeats was calculated on the electron densities of the 3D reconstructed filaments that were semi-automatically segmented with Amira which corresponds to threshold-based method. The computation was performed in Matlab using a non-linear least-squares regression algorithm nlinfit^28^. The animations showing the filament densities presented in the Supplementary Data have been generated with Amira.

### Bioinformatic analysis

TPR predictions were done using the TPRpred program^29^ and the secondary structure and closest structural homologues were inferred from Phyre 2^30^. The pdb entry 3CEQ of the Human kinesin light chain 2 was used to obtain the Bklc model with highest confidence over the largest sequence coverage with highest sequence identity and the model in Fig. 1B was done using Pymol (http://www.pymol.org). The amphipathic nature and hydrophobic characteristics of the N-terminal helix were predicted using the HELIquest software^31^.

Blast^32^ searches using Bklc from *Prosthecobacter vanneervenii* (Uniprot id: A8Y5U5_9BACT) as query were performed over several databases, including: Uniprot, non-redundant and IMG complete genomes and metagenomic databases. For all searches, an inclusion e-value threshold of 10^−10^ was used. Blast outputs showed a high rate of false positives, due in part to sequence divergence but also to variable number of TPR motifs associated with diverse protein functions. For this reason we turned to profile-based searches to search for eukaryotic homologues, using HHblits^33^ against the Uniprot database and HHsearch^34^ against the PDB. We also turned to transitive profile searches as implemented in HHsenser^35^ to explore distant homologues, as this method has proved to gather few false positive rates.

## Additional Information

**How to cite this article:** Akendengue, L. *et al*. Bacterial kinesin light chain (Bklc) links the Btub cytoskeleton to membranes. *Sci. Rep.* **7**, 45668; doi: 10.1038/srep45668 (2017).

**Publisher's note:** Springer Nature remains neutral with regard to jurisdictional claims in published maps and institutional affiliations.

## Supplementary Material

Supplemental Material

Supplemental Movie S1

Supplemental Movie S2

Supplemental Movie S3

Supplemental Movie S4

Supplemental Movie S5
