# Supplementary material for: Bacterial kinesin light chain (Bklc) links the Btub cytoskeleton to membranes
Source: Sci Rep. 2017 Mar 30;7:45668. doi: 10.1038/srep45668 (PMC5372463; doi:10.1038/srep45668)
Supplement: Supplemental Material [file srep45668-s1.pdf]

Supplemental Material

Bacterial kinesin light chain (Bklc) links the Btub cytoskeleton to membranes.

Lurlène Akendengue[1,2,8#], Sylvain Trépout[3,4#], Martín Graña[5], Alexis Voegele[6], Carsten Janke[1,2], Bertrand Raynal[7], Alexandre Chenal[6], Sergio Marco[3,4] & Anne Marie Wehenkel[1,2,9*]

1 Institut Curie, PSL Research University, CNRS UMR3348, F-91405 Orsay, France

2 Université Paris Sud, Université Paris-Saclay, CNRS UMR3348, F-91405 Orsay, France

3 INSERM, U1196, Université Paris Sud, Université Paris-Saclay, F-91405 Orsay, France

4 Institut Curie, PSL Research University, CNRS, UMR9187, F-91405 Orsay, France

5 Institut Pasteur Montevideo, Unidad de Bioinformática, Mataojo 2020, 11400 Montevideo, Uruguay

6 Institut Pasteur, Unité de Biochimie des Interactions Macromoléculaires, CNRS UMR3528, 28 rue du Dr Roux, 75724 Paris, France

7 Institut Pasteur, Plateforme de Biophysique Moléculaire, CNRS UMR3528, 28 rue du Dr Roux, 75724 Paris, France

8 Current address: Laboratoire de Biologie et Pharmacologie Appliquée, UMR8113 CNRS, Ecole Normale Supérieure Paris-Saclay, 94235 Cachan, France

9 Institut Pasteur, Unité de Microbiologie Structurale, CNRS UMR3528, Université Paris Diderot, 25 rue du Dr Roux, 75724 Paris, France

* Corresponding author: annemarie.wehenkel@pasteur.fr

# equal contribution

Supplemental Material and Methods

Size exclusion chromatography

The mixture containing BtubAB (26 μM) and Bklc (66 μM) were injected onto a Superdex S200 10/300 column at 4C. The collected fractions were run on a 12% SDS-PAGE.

Light scattering assay

Btub A/B assembly was monitored by right angle light scattering with a Hitachi spectrophotometer UV solutions U-2900, with wavelengths set at 350 nm. Purified Btub A/B was added to a final concentration 1.5, 2, 5, or 7.5 μM in the presence or absence of equimolar concentrations of Bklc in the appropriate polymerization buffer (300 mM KGlu, 5 mM $MgCl_2$, 1 mM EGTA, 20 mM TrisHCl pH7.5) to a quartz cuvette with a 1 cm pathlength. The cuvette was placed in a chamber that was maintained at 25°C and data were collected for 20 sec to establish a baseline followed by addition of 0.2 mM GTP for a final reaction volume of 500 μl. Data points were collected for 25 minutes in 3 or 2 independent measurements for Btubs alone or Btubs in the presence of Bklc respectively.

Supplemental Reference:

1. Schlieper, D., Oliva, M. A., Andreu, J. M. & Löwe, J. Structure of bacterial tubulin BtubA/B: evidence for horizontal gene transfer. *Proc Natl Acad Sci USA* **102,** 9170–9175 (2005).
2. Andreu, J. M. & Oliva, M. A. *Purification and Assembly*. *Microtubules, in vitro 2E* **115,** 269–281 (2013).
3. Sontag, C. A., Staley, J. T. & Erickson, H. P. In vitro assembly and GTP hydrolysis by bacterial tubulins BtubA and BtubB. *Journal of Cell Biology* **169,** 233–238 (2005).

Supplemental Figure Legends

Figure S1: Size exclusion chromatography of BtubA, BtubB and Bklc. The fractions corresponding to the extended part were run on an SDS-PAGE gel. The profile shows that the Btubs and Bklc elute as separate peaks and thus do not interact. BtubA and B

form weak heterodimers and elute as separate peaks as previously shown[1,2]. The light grey curve corresponds to the molecular weights markers at 158 kDa, 44 kDa, 13.5 kDa.

Figure S2: Polymerization of BtubA/B +/- Bklc followed by light scattering at 350 nm. The extrapolated intercept with the x-axis corresponds to the critical concentration of polymerization (0.45-0.77 μM) in accordance with the previously published values of 0.4-1.0 μM [3].

Movie Legends

Movie S1: Reconstructed 2-filament Btub assembly corresponding to Figure 3C left.

Movie S2: Reconstructed 4-filament Btub assembly corresponding to Figure 3C right.

Movie S3: Reconstructed 6-filament Btub assembly.

Movie S4: Reconstructed tomogram of BtubA+B fibers corresponding to Figure 3A.
Movie S5: Reconstructed tomogram of BtubA+B + Bklc fibers.

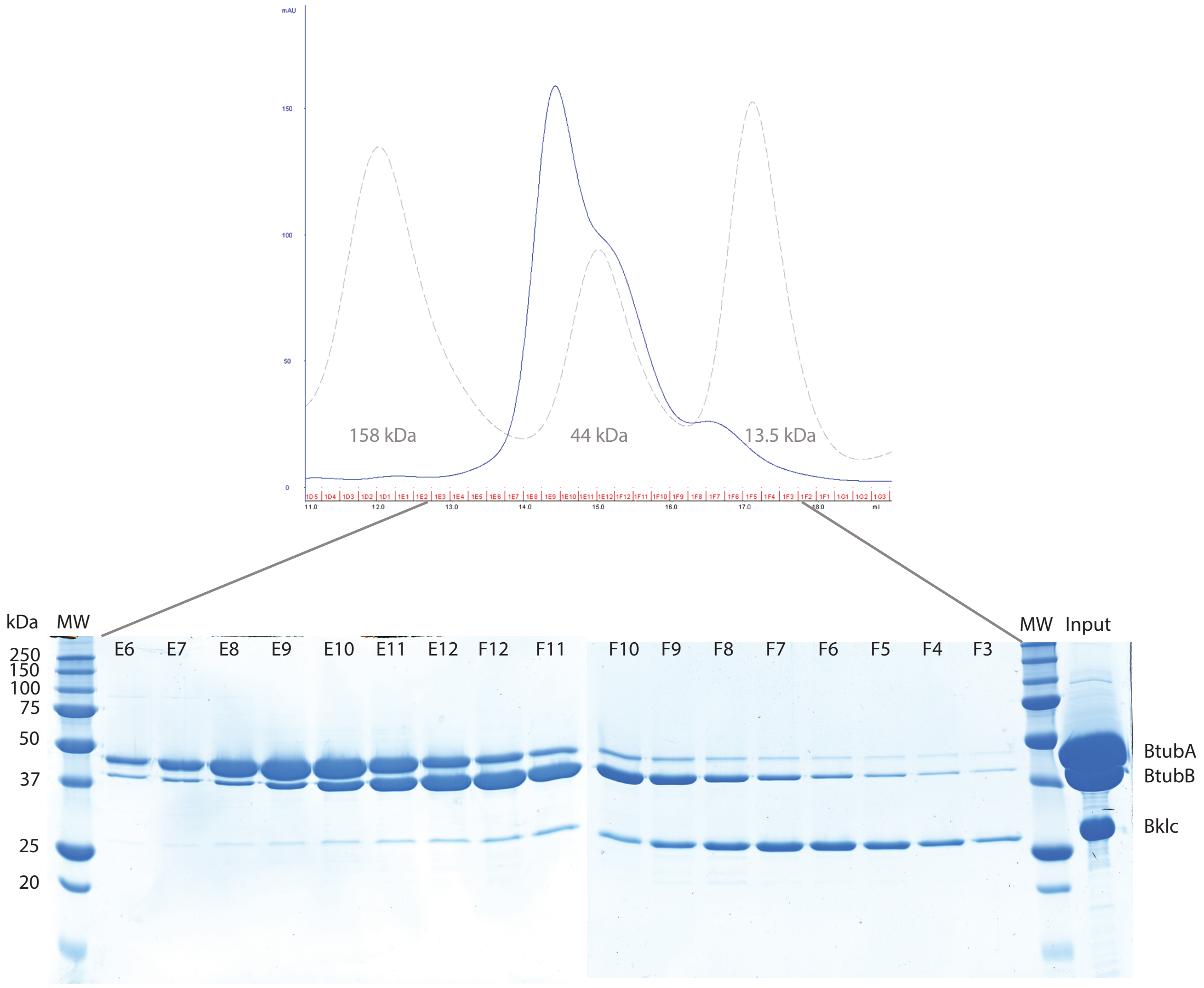

Supplementary Figure-S1

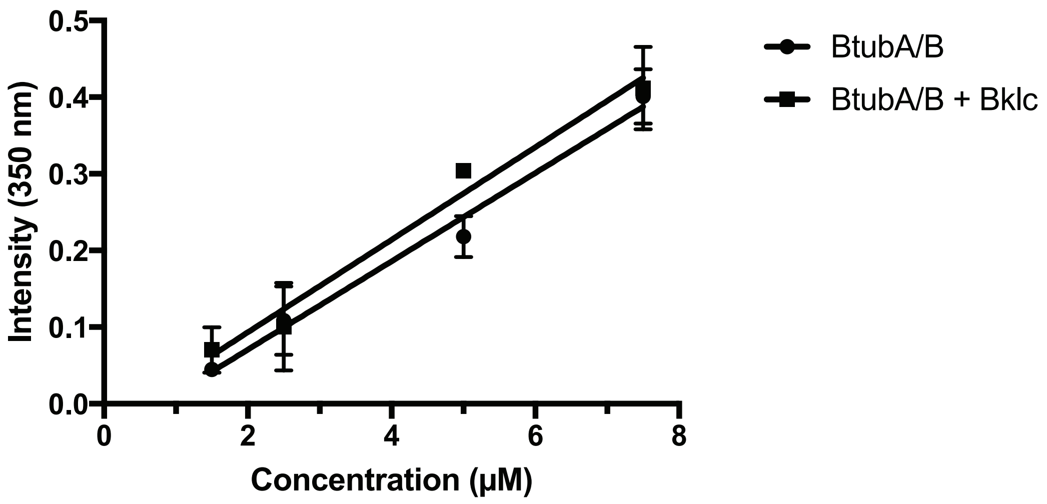

Supplementary Figure-S2
